# Effect of bisphosphonates or teriparatide on mechanical complications after posterior instrumented fusion for osteoporotic vertebral fracture: a multi-center retrospective study

**DOI:** 10.1186/s12891-020-03452-6

**Published:** 2020-07-01

**Authors:** Atsuyuki Kawabata, Toshitaka Yoshii, Takashi Hirai, Shuta Ushio, Takashi Kaito, Tomoya Yamashita, Hiroyasu Fujiwara, Yukitaka Nagamoto, Yuji Matsuoka, Hidekazu Suzuki, Hirosuke Nishimura, Hidetomi Terai, Koji Tamai, Atsushi Tagami, Syuta Yamada, Shinji Adachi, Kei Watanabe, Keiichi Katsumi, Masayuki Ohashi, Yohei Shibuya, Katsumi Harimaya, Kenichi Kawaguchi, Nobuhiko Yokoyama, Hidekazu Oishi, Toshiro Doi, Atsushi Kimura, Hirokazu Inoue, Gen Inoue, Masayuki Miyagi, Wataru Saito, Atsushi Nakano, Daisuke Sakai, Tadashi Nukaga, Shota Ikegami, Masayuki Shimizu, Toshimasa Futatsugi, Seiji Ohtori, Takeo Furuya, Sumihisa Orita, Shiro Imagama, Kei Ando, Kazuyoshi Kobayashi, Katsuhito Kiyasu, Hideki Murakami, Katsuhito Yoshioka, Shoji Seki, Michio Hongo, Kenichiro Kakutani, Takashi Yurube, Yasuchika Aoki, Masashi Oshima, Masahiko Takahata, Akira Iwata, Hirooki Endo, Tetsuya Abe, Toshinori Tsukanishi, Kazuyoshi Nakanishi, Kota Watanabe, Tomohiro Hikata, Satoshi Suzuki, Norihiro Isogai, Eijiro Okada, Haruki Funao, Seiji Ueda, Yuta Shiono, Kenya Nojiri, Naobumi Hosogane, Ken Ishii

**Affiliations:** 1 Department of Orthopaedic Surgery, Tokyo Medical and Dental University, 1-5-45 Yushima, Bunkyo-ku, Tokyo, 113-8519 Japan; 2 Department of Orthopaedic Surgery, Tokyo Medical and Dental University, 1 Chome-5-45 Yushima, Bunkyo City, Tokyo, 113-8510 Japan; 3 Department of Orthopaedic Surgery, Osaka University, 2-2 Yamadaoka, Suita City, Osaka 565-0871 Japan; 4 Department of Orthopaedic Surgery, Tokyo Medical University, 6-1-1 Shinjuku, Shinjuku-ku, Tokyo, 160-8402 Japan; 5 Department of Orthopaedic Surgery, Osaka City University, 1-4-3 Asahimachi, Abeno-ku, Osaka, 545-8585 Japan; 6 Department of Orthopaedic Surgery, Nagasaki University, 1-7-1 Sakamoto, Nagasaki City, Nagasaki 852-8501 Japan; 7 Department of Orthopedic Surgery, Niigata University Medical and Dental General Hospital, 1-757 Asahimachi Dori, Chuo-ku, Niigata City, Niigata 951-8510 Japan; 8 Department of Orthopaedic Surgery, Kyushu University, 3-1-1 Maidashi, Higashi-ku, Fukuoka City, 812-8582 Japan; 9 Department of Orthopaedic Surgery, Jichi Medical University, 3311-1 Yakushiji, Shimotsuke City, Tochigi 329-0498 Japan; 10 Department of Orthopaedic Surgery, Kitasato University, 1-15-1 Kitasato, Minami-ku, Sagamihara City, Kanagawa 252-0374 Japan; 11 Department of Orthopaedic Surgery, Osaka Medical College, 2-7 Daigakumachi, Takatsuki City, Osaka 569-0801 Japan; 12 Department of Orthopaedic Surgery, Tokai University, 143 Shimokasuya, Isehara City, Kanagawa 259-1193 Japan; 13 Department of Orthopaedic Surgery, Shinshu University, 3-1-1, Asahi, Matsumoto City, Nagano 390-8621 Japan; 14 Department of Orthopaedic Surgery, Chiba University, 1-8-1 Inohana, Chuo-ku, Chiba City, Chiba 260-8670 Japan; 15 Department of Orthopaedic Surgery, Nagoya University, 65 Tsurumai-cho, Showa-ku, Nagoya City, Aichi 466-8560 Japan; 16 Department of Orthopaedic Surgery, Kochi University, Oko-cho Kohasu, Nankoku City, Kochi 783-8505 Japan; 17 Department of Orthopaedic Surgery, Kanazawa University, 13-1 Takaramachi, Kanazawa City, Ishikawa 920-8641 Japan; 18 Department of Orthopaedic Surgery, University of Toyama, 2630 Sugitani, Toyama City, Toyama 930-0194 Japan; 19 Department of Orthopaedic Surgery, Akita University, 1-1-1 Hondo, Akita City, Akita 010-8543 Japan; 20 Department of Orthopaedic Surgery, Kobe University, 7-5-1 Kusunoki-cho, Chuo-ku, Kobe City, Hyogo 650-0017 Japan; 21 Department of Orthopaedic Surgery, Eastern Chiba Medical Center, 3-6-2 Okayamadai, Togane City, Chiba 283-8686 Japan; 22 Department of Orthopaedic Surgery, Nihon University Itabashi Hospital, 30-1 Oyaguchikamicho, Itabashi-ku, Tokyo, 173-8610 Japan; 23 Department of Orthopaedic Surgery, Hokkaido University, North-15, West-7, Kita-ku, Sapporo City, Hokkaido 060-8638 Japan; 24 Department of Orthopaedic Surgery, Iwate Medical University, 1-1-1 Idaidori, Yahaba-cho, Shiwa-gun, Iwate, 028-3694 Japan; 25 Department of Orthopaedic Surgery, University of Tsukuba, 1-1-1 Tennodai, Tsukuba City, Ibaraki 305-8577 Japan; 26 Department of Orthopaedic Surgery, Hiroshima University, 1-2-3 Kasumi, Minami-ku, Hiroshima City, Hiroshima 734-8551 Japan; 27 Department of Orthopaedic Surgery, Keio University School of Medicine, 35 Shinanomachi, Shinjuku-ku, Tokyo, 160-8582 Japan; 28 Department of Orthopaedic Surgery, School of Medicine, International University of Health and Welfare, Mita, Minato-ku, Tokyo, 108-8329 Japan; 29 Department of Orthopaedic Surgery, National Defense Medical College, 3-2 Namiki, Tokorozawa City, Saitama 359-8513 Japan

**Keywords:** Bisphosphonates, Teriparatide, Osteoporotic vertebral fractures, Surgery, Primary osteoporosis, Glucocorticoid-induced osteoporosis

## Abstract

**Background:**

The optimal treatment of osteoporosis after reconstruction surgery for osteoporotic vertebral fractures (OVF) remains unclear. In this multicentre retrospective study, we investigated the effects of typically used agents for osteoporosis, namely, bisphosphonates (BP) and teriparatide (TP), on surgical results in patients with osteoporotic vertebral fractures.

**Methods:**

Retrospectively registered data were collected from 27 universities and affiliated hospitals in Japan. We compared the effects of BP vs TP on postoperative mechanical complication rates, implant-related reoperation rates, and clinical outcomes in patients who underwent posterior instrumented fusion for OVF. Data were analysed according to whether the osteoporosis was primary or glucocorticoid-induced.

**Results:**

A total of 159 patients who underwent posterior instrumented fusion for OVF were included. The overall mechanical complication rate was significantly lower in the TP group than in the BP group (BP vs TP: 73.1% vs 58.2%, *p* = 0.045). The screw backout rate was significantly lower and the rates of new vertebral fractures and pseudoarthrosis tended to be lower in the TP group than in the BP group. However, there were no significant differences in lumbar functional scores and visual analogue scale pain scores or in implant-related reoperation rates between the two groups. The incidence of pseudoarthrosis was significantly higher in patients with glucocorticoid-induced osteoporosis (GIOP) than in those with primary osteoporosis; however, the pseudoarthrosis rate was reduced by using TP. The use of TP also tended to reduce the overall mechanical complication rate in both primary osteoporosis and GIOP.

**Conclusions:**

The overall mechanical complication rate was lower in patients who received TP than in those who received a BP postoperatively, regardless of type of osteoporosis. The incidence of pseudoarthrosis was significantly higher in patients with GIOP, but the use of TP reduced the rate of pseudoarthrosis in GIOP patients. The use of TP was effective to reduce postoperative complications for OVF patients treated with posterior fusion.

## Background

Osteoporotic vertebral fractures (OVFs) are the most common type of fragility fracture and are sustained by 25% of individuals in their early 70s and 43% of those aged over 80 years [1]. OVFs cause low back pain, which significantly impacts quality of life. Many cases can be cured by conservative treatment using a brace [2, 3], but surgery may be performed when early mobilization is desired, conservative treatment fails, or the posterior segment of the vertebra is destroyed, accompanied by neurological deficit [4]. Surgery is also an effective option for the treatment of kyphosis after fracture [5]. The recent advent of the percutaneous pedicle screw has made it possible to perform minimally invasive surgery in these patients [6]. However, because of the bone fragility in the background, surgically treated patients may experience screw loosening or pseudoarthrosis and revision surgery may be required [7].

BP are the first-line agents for osteoporosis and have been shown to prevent fragility fractures by inhibiting osteoclasts [8]. TP is a recombinant form of human parathyroid hormone and is the first osteogenic agent to enter clinical use. Improvements in lumbar bone mineral density (BMD) of 4.54–7.48% have been reported for BP, whereas 6.4% improvement of BMD was reported after 2 years of administration of TP [9]. However, because TP is expensive and the allowed duration of administration is limited, the use of TP is problematic in terms of timing [10]. In contrast, several studies have reported osteonecrosis of the jaw or atypical femoral fracture as adverse effects of BP [11–13], which are not observed under TP.

Previous studies have demonstrated the superiority of TP over BP in patients who have undergone conservative treatment for OVF according to bone union rates [14–16]. Other studies have shown the positive effects of TP on bony fusion after spinal fusion or correction surgery [17–22]. TP has bone anabolic ability and can reduce the chance of screw loosening, and so the use of TP may effectively enhance spinal fusion and reduce implant failure and/ or subsequent vertebral fracture in comparison with surgery [17–22]. However, there is limited literature on the relationship between administration of these agents and mechanical complications after posterior fusion surgery for OVF. Furthermore, few studies have investigated the effects of TP and BP in patients with glucocorticoid-induced osteoporosis (GIOP). Therefore, optimal treatment of osteoporosis after reconstruction surgery for OVF remains unclear. The aim of this nationwide multicentre retrospective study was to investigate the effects of these osteoporosis medications on surgical outcomes, specifically mechanical complications and reoperation rates in patients with primary OVF or GIOP.

## Methods

### Data collected

We retrospectively reviewed all patients who were treated surgically with instrumentation for OVF between 2005 and 2014 at 27 universities and affiliated hospitals that contribute data to the multicentre Japan Association of Spine Surgeons with Ambition (JASA)database. Institutional review board approval was obtained at each site for patient enrolment and the data collection protocols. The need for informed consent was waived by the ethics committees because this retrospective cohort study involved existing data and records at the time of investigation, and did not retain personal identifiers in the collected information.

### Inclusion criteria

Inclusion criteria were patients who had neurological impairments due to OVF at T10-L5 and underwent posterior fusion surgery. The surgical indication was any neurological symptoms caused by OVF and related neural compression, which required stabilization. Patients who underwent vertebroplasty alone were not included. To focus on comparison of effect of BP and TP on the surgical outcomes, we only included the most typical surgical procedures: posterior fusion with fusion levels less than 5. Patients who received treatment with a BP or TP (≥ 6 months) after surgery were included. The patients were followed for average of 3.9 years. TP was administered for up to 2 years (20 μg/day, average: 17.5 months) and then generally followed by a BP for the purpose of secondary mineralization during the follow-up period. Regarding the BP treatment, The patients were treated with either alendronate (5 mg/day or 35 mg/week), residronate (2.5 mg/day or 17.5 mg/week), ibandronate (100 mg/month) or minodronate (50 mg/month). Patients who received other osteoporosis medications, such as denosumab or a selective oestrogen receptor modulator, were excluded. The aetiology was primary osteoporosis or GIOP. The patients who had the history of diseases causing secondary osteoporosis, such as hypothyroidism, rheumatoid arthritis, diabetes mellitus, and chronic renal failure were excluded. The operative procedure in the database was divided into anterior spinal fusion, posterior spinal fusion alone with or without vertebroplasty, and anterior and posterior three-column osteotomy. Patients who underwent posterior spinal fusion fixation for less than five levels were included in this study. Demographic data including age, sex, body mass index, BMD and preoperative and postoperative use of osteoporosis agents were collected. BMD was expressed as a percentage of the young adult mean (aged from 20 to 44 years). YAM less than 70% was used to diagnose osteoporosis in Japan [23].

### Analysis of data

Radiological examination was performed by X-ray. The data of preoperative, postoperative (early after surgery) and final follow-up were collected. Postoperative mechanical complications were defined as screw loosening, screw backout, screw breakage, rod breakage, and subsequent vertebral fractures (newly developed fracture after surgery) [18, 24]. Screw loosening and screw backout are common complications in spinal instrumentation surgeries. Loosening of screw was detected by radiolucent zones around the screw indicating a low extraction torque. Screw back out was defined as change in screw position due to screw loosening. Screw complication was defined as screw loosening, screw backout, and screw breakage. Pseudoarthrosis was defined as one of following at the final follow-up: instability with flexion/extension radiographs, presence of clear zone around the bone graft, or rod fracture. Clinical outcomes were evaluated using the Japanese Orthopaedic Association (JOA) scoring system for lumbar function [25, 26] preoperatively and at the final follow-up. For JOA score, only scores for subjective symptoms (9 points), clinical signs (6 points), and urinary bladder function (− 6 points) were included; a full score was 15 points (Additional file 1). The visual analogue scale (VAS) score was used to grade the severity of low back pain and pain or numbness in the lower extremities (0, no symptoms; 10, worst symptoms imaginable). The recovery rate of JOA was calculated using following formula: recovery rate (%) = (postoperative score (final follow-up) − preoperative score)/(15 − preoperative score) × 100 [25]. The change in VAS was defined as reduction of VAS between preoperative and postoperative score. The patients were divided into those who received BP and those who received TP, postoperatively. We compared the patient’s demographics and surgical outcomes, as well as postoperative complications between the BP and TP group. Further, we compared the effect of BP and TP for patients with primary osteoporosis and GIOP.

### Statistics

The *t*-test or chi-squared test was used to compare the two groups. The averages of continuous variables were compared between the groups using *t*-tests and the proportions of categorical variables were compared using chi-square or Fisher’s exact tests. All statistical analyses were performed using IBM SPSS Statistics for Macintosh software version 25.0 (IBM Corp., Armonk, NY). A *p*-value < 0.05 was considered statistically significant.

## Results

One hundred and fifty-nine patients who underwent posterior instrumented fusion during the study period met the inclusion criteria. The mean patient age at the time of surgery was 75.6 ± 6.7 years and 136 patients (85.5%) were female. The mean YAM score was 71.0 ± 16.1%. The OVFs were caused by primary osteoporosis in 132 patients and by GIOP in 27 patients. The number of previous vertebral fracture was 0.5 ± 0.9 and the number of new vertebral fracture causing fusion surgery was 1.1 ± 0.2. The time from injury to fusion surgery was 173 ± 240 days.

Table 1 shows the patient characteristics according to type of osteoporosis. Mean patient age at the time of surgery was significantly higher in the primary osteoporosis group than in the GIOP group (primary vs GIOP: 76.6 ± 6.4 years vs 71.0 ± 6.1 years; *p* = 0.001). Significantly more patients in the GIOP group received an osteoporosis medication preoperatively (*p* < 0.001). However, there was no significant between-group difference in sex, body mass index, smoking history, number of vertebral fractures, time from injury to surgery, BMD, and number of fixed levels. The preoperative JOA and VAS scores for back pain and leg symptoms were also similar between the two groups.

**Table 1:** Preoperative demographic and clinical characteristics according to whether osteoporosis was primary or glucocorticoid-induced

	Total	Primary	GIOP	***p***-value
Cases, n	159	132	27	–
Age at time of surgery (years)	75.6 ± 6.7	76.6 ± 6.4	71.0 ± 6.1	0.001
Sex (male)	136 (85.5%)	111 (84.1%)	25 (92.6%)	0.37
BMI	22.3 ± 5.1	22.4 ± 4.7	22.3 ± 6.4	0.92
Smoking history (yes/no)	20 (12.6%)	19 (14.4%)	1 (3.7%)	0.20
Number of previous vertebral fracture	0.5 ± 0.9	0.4 ± 0.9	0.7 ± 1.2	0.20
Number of new vertebral fracture	1.1 ± 0.2	1.1 ± 0.2	1.1 ± 0.3	0.79
Time from injury to surgery (days)	173 ± 240	169 ± 251	192 ± 184	0.65
BMD (YAM%)	71.0 ± 16.1	70.4 ± 14.5	73.5 ± 16.4	0.55
Preoperative use of osteoporosis medication	71 (44.7%)	49 (37.1%)	22 (81.5%)	< 0.001
BP	55 (34.6%)	41 (31.1%)	14 (51.9%)	0.047
Vit D	14 (8.8%)	7 (5.3%)	7 (25.9%)	0.003
TP	7 (4.4%)	4 (3.0%)	3 (11.1%)	0.10
Number of fixed level	3.1 ± 0.8	3.1 ± 0.8	3.0 ± 0.9	0.53
Preoperative JOA score	4.2 ± 3.4	4.3 ± 3.3	3.4 ± 3.9	0.27
Preoperative VAS (back pain)	71.5 ± 24.4	70.7 ± 24.3	75.3 ± 24.9	0.39
Preoperative VAS (leg pain or numbness)	55.0 ± 31.7	55.0 ± 31.9	54.7 ± 31.5	0.696

*GIOP* glucocorticoid-induced osteoporosis, *BMI* body mass index, *BMD* bone mineral density, *YAM* young adult mean, *BP* bisphosphonate, *Vit D* vitamin D, *TP* teriparatide, *JOA* Japan Orthopaedic Association, *VAS* visual analogue scale

The demographic characteristics in the BP and TP groups are shown in Table 2. There was no significant difference in age, sex, body mass index, BMD, smoking status, number of vertebral fractures, time from injury to surgery, or the preoperative JOA or VAS scores between the two groups. Preoperative use of osteoporosis medication was significantly more common in the TP group than in the BP group (BP vs TP: 36.5% vs 60.0%, *p* = 0.007). There was no significant difference in the number of fixed levels between the two groups.

**Table 2:** Preoperative demographic and clinical characteristics according to whether osteoporosis medication was BP or TP

	BP	TP	***p***-value
Cases, n	104	55	–
Age at surgery, years	75.2 ± 6.8	76.4 ± 6.4	0.46
Sex (female)	89 (85.6%)	47 (85.5%)	0.98
BMI	22.2 ± 4.8	22.6 ± 5.7	0.62
Smoking history	14 (13.5%)	6 (10.9%)	0.64
Number of previous vertebral fractures	0.4 ± 0.89	0.6 ± 1.0	0.20
Number of new vertebral fracture	1.0 ± 0.2	1.1 ± 0.3	0.13
Time from injury to surgery (days)	183 ± 275	152 ± 154	0.44
BMD, YAM%	72.5 ± 17.7	73.0 ± 13.9	0.87
Preoperative drug use for osteoporosis	38 (36.5%)	33 (60.0%)	0.007
BP	32 (30.8%)	23 (41.8%)	0.22
Vit D	7 (6.7%)	7 (12.7%)	0.24
TP	0	7 (12.7%)	< 0.001
Number of fixed levels	3.0 ± 0.8	3.2 ± 0.8	0.21
Preoperative JOA score	4.8 ± 3.6	4.1 ± 3.3	0.15
Preoperative VAS (back pain)	71.2 ± 22.9	74.6 ± 27.1	0.25
Preoperative VAS (leg pain or numbness)	53.1 ± 31.6	58.4 ± 32.9	0.36

*BP* bisphosphonate, *TP* teriparatide, *BMI* body mass index, *BMD* bone mineral density, *YAM* young adult mean, *Vit D* vitamin D, *JOA* Japanese Orthopaedic Association, *VAS* visual analogue scale

Table 3 shows the postoperative complications and clinical outcomes in the BP and TP groups. The overall mechanical complication rate was significantly higher in the BP group than in the TP group (BP vs TP: 73.1% vs 58.2%, *p* = 0.045). The screw backout rate was significantly lower and the rates of development of new vertebral fractures and pseudoarthrosis tended to be lower in the TP group than in the BP group (BP vs TP: screw backout, 12.5% vs 1.8%; vertebral fracture, 45.2% vs 32.7%; pseudoarthrosis, 8.7% vs 1.8%). There were no significant differences in the rates of revision surgery or other complications, such as screw loosening and rod fracture, between the groups. Furthermore, there were no significant differences in the postoperative JOA and VAS scores or in their rates of improvement.

**Table 3:** Postoperative complications and clinical outcomes according to whether osteoporosis medication was BP or TP

	BP	TP	p-value
Cases, n	104	55	–
Mechanical complications	76 (73.1%)	32 (58.2%)	0.045
Subsequent vertebral fracture	47 (45.2%)	18 (32.7%)	0.18
Screw complications	42 (40.4%)	19 (34.5%)	0.29
Screw loosening	29 (27.9%)	18 (32.7%)	0.59
Screw backout	13 (12.5%)	1 (1.8%)	0.024
Rod fracture	0	0	–
Pseudoarthrosis	9 (8.7%)	1 (1.8%)	0.091
Revision due to mechanical complications	10 (9.6%)	9 (16.4%)	0.30
Postoperative JOA	9.7 ± 3.4	9.3 ± 3.3	0.45
Recovery rate of JOA	49.0 ± 27.5	52.1 ± 25.2	0.49
Final VAS (back pain)	29.8 ± 22.5	31.3 ± 24.5	0.71
Change in VAS (back pain)	40.4 ± 30.0	41.6 ± 32.3	0.83
Final VAS (leg pain or numbness)	18.4 ± 20.4	21.2 ± 25.3	0.46
Change in VAS (leg pain or numbness)	33.5 ± 27.1	35.8 ± 30.1	0.63

*BP* bisphosphonate, *TP* teriparatide, *JOA* Japanese Orthopaedic Association, *VAS* visual analogue scale

Table 4 shows postoperative complications and clinical outcomes compared between the patients with primary osteoporosis and those with GIOP. The rate of pseudoarthrosis was significantly higher in patients with GIOP than in those with primary osteoporosis (primary vs GIOP: 4.3% vs 14.8%, *p* = 0.045). Other complications were not significantly different between the two groups. There were no significant differences in postoperative JOA and VAS scores or the recovery rates.

**Table 4:** Postoperative complications and clinical outcomes according to whether osteoporosis was primary or glucocorticoid-induced

	Primary	GIOP	***p***-value
Cases, n	132	27	–
Mechanical complications	91 (67.4%)	17 (63.0%)	0.54
New vertebral fracture	57 (42.8%)	8 (29.6%)	0.21
Screw complications	54 (39.1%)	7 (25.9%)	0.21
Screw loosening	42 (30.4%)	5 (18.5%)	0.25
Screw backout	12 (8.7%)	2 (7.4%)	0.78
Rod fracture	0	0	–
Pseudoarthrosis	6 (4.3%)	4 (14.8%)	0.045
Revision due to mechanical complications	17 (12.3%)	2 (7.4%)	0.43
Postoperative JOA	9.7 ± 3.5	8.8 ± 3.0	0.21
Recovery rate of JOA	50.7 ± 27.8	46.1 ± 20.1	0.45
Final VAS (back pain)	30.0 ± 23.7	32.1 ± 20.6	0.74
Change in VAS (back pain)	40.3 ± 30.9	39.4 ± 29.8	0.72
Final VAS (leg pain or numbness)	19.2 ± 22.1	22.3 ± 23.4	0.79
Change in VAS (leg pain or numbness)	34.2 ± 29.0	34.8 ± 23.3	0.99

*BP* bisphosphonate, *TP* teriparatide, *JOA* Japanese Orthopaedic Association, *VAS* visual analogue scale

Table 5 shows the postoperative complications and clinical outcomes in the BP and TP groups according to whether the cause of OVF was primary osteoporosis or GIOP. The overall mechanical complication, screw backout, and pseudoarthrosis rates tended to be reduced by the use of TP compared to the use of BP in patients with primary osteoporosis (overall mechanical complications, BP vs TP: 72.0% vs 61.5%, *p* = 0.16; screw backout, 11.8% vs 2.6%, *p* = 0.080; pseudoarthrosis, 6.5% vs 0%, *p* = 0.12). In patients with GIOP, the rates of these complications were more markedly reduced by using TP (overall complications, 81.8% vs 50%, *p* = 0.093; screw backout, 18.2% vs 0% *p* = 0.076; pseudoarthrosis, 27.3% vs 6.3%, *p* = 0.13). The screw loosening and revision surgery rates were not different between the BP and TP groups in both types of osteoporosis. There were no significant differences in the postoperative JOA and VAS scores or their recovery rates.

**Table 5:** Postoperative complications and clinical outcomes in the BP and TP groups according to whether the cause of OVF was primary osteoporosis or GIOP

	Primary osteoporosis			GIOP		
	BP	TP	***p***-value	BP	TP	***p***-value
Cases, n	93	39	–	11	16	–
Mechanical complications	67 (72.0%)	24 (61.5%)	0.16	9 (81.8%)	8 (50%)	0.093
New vertebral fracture	42 (45.2%)	15 (38.5%)	0.30	5 (45.5%)	3 (18.8%)	0.14
Screw complications	39 (41.9%)	15 (38.5%)	0.43	3 (27.3%)	4 (25.0%)	0.90
Screw loosening	28 (30.1%)	14 (35.9%)	0.54	1 (9.1%)	4 (25.0%)	0.30
Screw backout	11 (11.8%)	1 (2.6%)	0.08	2 (18.2%)	0	0.076
Rod fracture	0	0	–	0	0	–
Pseudoarthrosis	6 (6.5%)	0	0.12	3 (27.3%)	1 (6.3%)	0.13
Revision due to mechanical complications	10 (10.8%)	7 (17.9%)	0.27	0	2 (12.5%)	0.50
Postoperative JOA score	9.9 ± 3.3	9.6 ± 3.7	0.75	9.3 ± 3.2	8.3 ± 3.0	0.49
JOA score recovery rate	49.2 ± 30.1	54.4 ± 25.9	0.32	48.0 ± 19.8	44.7 ± 26.3	0.69
Final VAS (back pain)	30.9 ± 23.7	29.8 ± 26.0	0.95	27.2 ± 20.1	36.2 ± 20.3	0.30
Change in VAS (back pain)	40.5 ± 30.9	40.3 ± 32.1	0.98	40.3 ± 26.7	38.7 ± 33.6	0.90
Final VAS (leg pain or numbness)	18.9 ± 21.4	19.9 ± 24.8	0.81	15.0 ± 14.7	28.9 ± 28.2	0.15
Change in VAS (leg pain or numbness)	34.7 ± 28.9	34.9 ± 31.9	0.87	29.2 ± 20.9	39.9 ± 25.1	0.28

*BP* bisphosphonate, *TP* teriparatide, *GIOP* glucocorticoid-induced osteoporosis, *JOA* Japanese Orthopaedic Association, *VAS* visual analogue scale

## Discussion

Previous studies have demonstrated that TP has a higher union rate in patients with OVF who were treated conservatively compared with those treated with BP [14–16]. However, little is known about the effect of these agents in patients with surgically treated OVF. Furthermore, there has been no comparison of the surgical outcomes between patients with primary osteoporosis and those with GIOP. In this study, we compared the surgical complications and clinical outcomes between patients with primary osteoporosis and GIOP. We then compared the effects of postoperative administration of teriparatide on surgical complications and reoperation rates with those of BP in patients with primary osteoporosis and in patients with GIOP.

The demographic data showed that patients with GIOP were younger at the time of surgery than those with primary osteoporosis. This finding indicates that vertebral fractures can occur at a relatively younger age in patients with GIOP. Consistent with this result is a review describing vertebral fractures occurring at a relatively young age in patients with GIOP, and the severity of the collapse was unexpectedly disproportionate to the age of the patients [27]. In our study, patients with GIOP had a relatively higher BMD than those with primary osteoporosis. A previous review also reported that patients on glucocorticoids experienced fractures at a higher BMD than the general population (i.e., the “BMD and bone fragility paradox”) [28]. The effect of glucocorticoids on bone is mediated by multiple pathophysiologic mechanisms resulting in an inhibition of bone formation and an increase in bone resorption. Glucocorticoids also impair the function of osteocytes and thus lead to impaired bone architecture [27] [28]. Saito et al. reported that deterioration of bone material properties advances with glucocorticoid use and the resulting alterations in bone quality are not captured by BMD [29]. Thus, dual-energy X-ray absorptiometry may underestimate the risk of fracture, which is estimated to be high even though the value of BMD is relatively high in patients treated with glucocorticoids.

In this study, the screw complications including screw loosening and screw back-out were recognized in 61/159 cases (38.4%) and non-union was found in 10/159 cases (6.3%). Previously, Uchida et al. evaluated the clinical outcomes of fusion surgeries for OVF with neurological impairments [30]. They reported that implant failure was most commonly observed complications either in anterior fusion (21.4%) and posterior fusion (25.0%). Non-union was recognized in 7.1% in anterior fusion and 8.3% in posterior fusion. A recent study demonstrated that screw complications were found in 18/38 patients (47.4%) in posterior fusion for OVFs [31]. Subsequent vertebral fracture after surgery was found in 65/159 (40.9%) in this study, which is similar to the previously reported incidence (38.3%) in posterior fusion for OVF with neurological deficits [18]. The incidences of such mechanical complications varied among the reports, but the incidences were clearly high in any reports and needed to be reduced in order to improve the surgical results.

Our study demonstrated that the use of PTH had positive effect to reduce the mechanical complications. The overall mechanical complication and screw backout rates were significantly lower and the new vertebral fracture and pseudoarthrosis rates tended to be lower by TP admiration for more than 6 months (average: 17.5 months). Ohtori et al. demonstrated that the use of TP (20 μg/day) for 12 months reduced screw loosening in 1- or 2-level posterior fusion for osteoporotic patients with lumbar degenerative diseases [21]. Ohtori et al. also reported that the use of TP for more than 6 months was more effective for union after lumbar fusion comparted with patients who received TP injection for less than 6 months [22]. Seki et al. reported that the use of TP (20 μg/day) for 24 months reduced adjacent vertebral fracture, implant failure, and fusion failure rates, compared with BP treatment, in correction surgeries for adult spinal deformity [17]. Therefore, TP has a bone anabolic effect and has effects on both bone fusion promotion and prevention of screw loosening [17, 21, 22]. Furthermore, the use of TP also reduces the risk of subsequent fractures in OVF patients as our study and previous studies demonstrated [17]. In accordance with these studies, our result also suggested that TP injection (≥ 6 months) may be considered useful for reducing mechanical complications and pseudoarthrosis in surgically treated cases of OVF.

In the comparison of primary and steroid-induced osteoporosis, our study showed that the rate of pseudoarthrosis was significantly higher in the patients with GIOP than in those with primary osteoporosis. Previous studies have reported that systemic corticosteroid therapy interfered with bony healing in a rabbit model, with a 60% lower union rate in the steroid group [32, 33]. Although there have been no reports demonstrating that the use of glucocorticoid is associated with pseudoarthrosis in human, our results suggested that glucocorticoid use hindered bony union in patients who underwent posterior spinal fusion. However, our study also demonstrated a favourable effect of TP compared with BP for prevention of pseudoarthrosis in patients with GIOP. Thus, administration of TP may be a viable option to improve the rate of bony healing especially for patients with GIOP.

We found that TP tended to reduce mechanical complications and pseudoarthrosis regardless of whether osteoporosis was primary or glucocorticoid-induced when compared with BP. Furthermore, the effect of TP on prevention of new vertebral fractures was stronger in our patients with GIOP than in those with primary osteoporosis. Saag et al. have demonstrated that TP is an effective treatment for GIOP and that it was superior to alendronate for prevention of vertebral fractures at 36 months (1.7% vs 7.7%) [34]. In accordance with the previous study, we found that TP had a positive effect on the mechanical complication rate. However, there has been a suggestion that the effect of TP is attenuated when the glucocorticoid dosage is higher than 15 mg/day [28]. Only four patients in our study were on glucocorticoid doses higher than 15 mg/day, thus a further study is needed to clarify the association between high-dose glucocorticoids and the effect of TP.

Previous studies have found better quality of life and VAS scores for back pain in patients treated with TP than in those treated with BP. In a crossover study by Jakob et al., postmenopausal women with osteoporosis who switched from a BP to TP reported better health-related quality of life and had improved VAS scores for back pain [14]. In our study, despite the positive effect of TP on mechanical complications, postoperative JOA and VAS scores for back pain were not significantly different between the TP and BP group. A possible explanation for this result is that posterior fusion itself results in marked improvement in the VAS score and quality of life. Therefore, symptoms in patients who received BP were also sufficiently improved by surgery, such that the effect of TP may not have been significant. However, the difference in mechanical complication rates between TP and BP may further increase during long term follow-up, which may result in marked differences in pain and quality of life in the patients with spinal fusion for OVFs.

This study has several limitations. First, we did not evaluate the type of BP and the exact duration of drug administration. Second, the rate of preoperative usage of osteoporosis medication was different between primary osteoporosis and GIOP group and BP and TP group. Third, we did not evaluate the surgeon’s experience and the tendency of each surgeon’s choice of osteoporosis treatment. Fourth, the number of GIOP patients was relatively small. Further studies with more appropriate study design with a large sample size are needed.

## Conclusions

In this study, the effect of postoperative TP on the overall mechanical complication rate was more favourable than that of a BP regardless of whether the osteoporosis is primary or glucocorticoid-induced. The incidence of pseudoarthrosis was significantly higher in patients with GIOP, but the use of TP reduced the rate of pseudoarthrosis in GIOP patients. The use of TP was effective to reduce postoperative complications for OVF patients treated with posterior fusion.

## Supplementary information

**Additional file 1.** Modified Japanese Orthopaedic Association (JOA) scoring system for lumbar function.

## Data Availability

The datasets generated during and/or analysed during this study are available from the corresponding author on reasonable request.

## Abbreviations

BMD: Bone mineral density
BP: Bisphosphonate
GIOP: Glucocorticoid-induced osteoporosis
JOA: Japanese Orthopaedic Association
OVF: Osteoporotic vertebral fracture
TP: Teriparatide
VAS: Visual analogue scale
YAM: Young Adult Mean
