# Supplementary material for: Effect of bisphosphonates or teriparatide on mechanical complications after posterior instrumented fusion for osteoporotic vertebral fracture: a multi-center retrospective study
Source: BMC Musculoskelet Disord. 2020 Jul 1;21:420. doi: 10.1186/s12891-020-03452-6 (PMC7331246; doi:10.1186/s12891-020-03452-6)
Supplement: Supplementary file 1 — Additional file 1. Modified Japanese Orthopaedic Association (JOA) scoring system for lumbar function. [file 12891_2020_3452_MOESM1_ESM.docx]

Additional file 1 Modified Japanese Orthopaedic Association (JOA) scoring system for lumbar function

Score

Subjective symptoms (9 points)

Low back pain

None 3

Occasionally mild 2

Always present or occasionally severe 1

Always severe 0

Leg pain/tingling

None 3

Occasionally mild 2

Always present or occasionally severe 1

Always severe 0

Ability to walk

Normal 3

~500m 2

500m~ 1

At most 100m 0

Objective findings (6 points)

SLR (including hamstring tightness)

Normal 2

30-70 degree 1

<30 degree 0

Sensory disturbance

Normal 2

Mild 1

Severe 0

MMT

Normal 2

Slight decrease 1

Marked decrease 0

Bladder and bowel dysfunction (BBD)(-6points)

Normal 0

Mild -3

Severe -6

Total score 15 points

MMT indicates manual muscle test; SLR, straight leg raising test.
